# CPAP Treatment Exposure, but Not Daytime Sleepiness or Neurofilament Light Chain, Is Associated with Cognitive Performance in Obstructive Sleep Apnea

**DOI:** 10.3390/jcm15041588

**Published:** 2026-02-18

**Authors:** Sofia Tagini, Stefania Cattaldo, Federica Scarpina, Erica Sabattini, Giulia Chirchio, Elisa Prina, Paolo Piterà, Clara Paschino, Riccardo Cremascoli, Mirna Solange Barrio Lower Daniele, Mauro Cornacchia, Theodore Tsaras, Amelia Brunani, Massimo Scacchi, Paolo Fanari, Alessandro Mauro, Lorenzo Priano

**Affiliations:** 1 “Rita Levi Montalcini” Department of Neurosciences, University of Turin, 10126 Turin, Italy; sofia.tagini@unito.it (S.T.); giulia.chirchio@unito.it (G.C.); e.prina@auxologico.it (E.P.); p.pitera@auxologico.it (P.P.); alessandro.mauro@unito.it (A.M.); l.priano@auxologico.it (L.P.); 2 Laboratory of Clinical Neurobiology, IRCCS Istituto Auxologico Italiano, San Giuseppe Hospital, 28824 Piancavallo, Italy; s.cattaldo@auxologico.it (S.C.); e.sabattini@auxologico.it (E.S.); c.paschino@auxologico.it (C.P.); 3 U.O. di Neurologia e Neuroriabilitazione, IRCCS Istituto Auxologico Italiano, San Giuseppe Hospital, 28824 Piancavallo, Italy; r.cremascoli@auxologico.it; 4 U.O. Medicina Generale, IRCCS Istituto Auxologico Italiano, San Giuseppe Hospital, 28824 Piancavallo, Italy; m.solange@auxologico.it (M.S.B.L.D.); massimo.scacchi@unimi.it (M.S.); 5 U.O. di Riabilitazione Pneumologica, IRCCS Istituto Auxologico Italiano, San Giuseppe Hospital, 28824 Piancavallo, Italy; m.cornacchia@auxologico.it (M.C.); p.fanari@auxologico.it (P.F.); 6 U.O. di Medicina Riabilitativa, IRCCS Istituto Auxologico Italiano, San Giuseppe Hospital, 28824 Piancavallo, Italy; t.tsaras@auxologico.it (T.T.); brunani@auxologico.it (A.B.); 7 Department of Clinical Sciences and Community Health, Dipartimento di Eccellenza 2023-2027, University of Milan, 20122 Milan, Italy

**Keywords:** obstructive sleep apnea syndrome, OSAS, cognition, cognitive impairment, verbal memory, problem-solving, sleepiness, neurofilament light chain, biomarkers

## Abstract

**Background:** The mechanisms underlying cognitive impairment in obstructive sleep apnea syndrome (OSAS) remain incompletely understood. In particular, the relative contribution of daytime sleepiness versus the direct effects of hypoxia on the brain requires clarification. **Objectives:** This study aims to explore the association between verbal memory and problem-solving abilities, OSAS severity, self-reported daytime sleepiness, and neurofilament light chain (NfL) serum concentration, as marker of neuroaxonal injury possibly related to chronic hypoxia. **Methods:** In this cross-sectional study, cognitive performance was assessed in 72 patients with mild to severe OSAS using the Selective Reminding Test (SRT) and the Tower of London (ToL). The apnea–hypopnea index (AHI), the Epworth Sleepiness Scale (ESS) and serum NfL concentrations were collected. Hierarchical multiple linear regression analyses adjusting for age, years of education, body mass index, and duration of continuous positive airway pressure (CPAP) treatment, were conducted for each cognitive outcome. **Results**. Neither AHI, ESS scores nor serum NfL concentrations were significantly associated with verbal memory or problem-solving performance. Higher age and lower education were significant predictors of lower verbal memory indices, whereas longer CPAP treatment duration was positively correlated with verbal memory performance and problem-solving speed. **Conclusions:** In this sample, cognitive performance was more strongly related to demographic factors and CPAP exposure compared to disease severity, subjective sleepiness, or peripheral markers of neuroaxonal injury. These findings suggest that AHI, subjective measures of sleepiness and NfL may inadequately capture neurocognitive vulnerability in OSAS. Moreover, they underscore the imperative for longitudinal and larger studies to clarify mechanisms linking OSAS and cognitive impairments.

## 1. Introduction

Obstructive Sleep Apnea Syndrome (OSAS) is a breathing disorder characterized by recurrent episodes of partial or complete upper airway obstruction during sleep. These events induce intermittent hypoxemia and sleep fragmentation, commonly resulting in excessive daytime sleepiness [1,2,3]. Beyond its recognized effects on quality of life and mood [4,5], accumulating evidence indicates that the pathophysiological features of OSAS exert deleterious effects also on cognition. Impairment in attention and vigilance, memory, visuospatial abilities, and executive functions has been consistently reported in OSAS [6,7,8]; however, inter-individual variability [9,10] suggests that at least two different mechanisms may interact to shape cognitive outcomes in affected patients [11,12,13]. Excessive daytime sleepiness has been shown to influence cognitive performance in OSAS, with attentional and memory deficits appearing more closely related to reduced daytime vigilance [14]. In contrast, executive dysfunction and global cognitive decline may be more strongly associated with the detrimental effects of nocturnal hypoxemia on the brain [15]. Chronic intermittent hypoxia and sleep fragmentation may synergistically promote cerebral hypoperfusion, endothelial dysfunction, neuroinflammation, and blood–brain barrier disruption; accordingly, oxidative stress, autonomic dysregulation, impaired cerebrovascular reactivity, and reduced glymphatic clearance of neurotoxic proteins might favor neurodegenerative processes [16,17,18,19,20,21].

In this study, we aimed to contribute to the ongoing debate on the mechanisms underlying cognitive impairment in OSAS by examining the association between cognitive performance, OSAS severity, self-reported daytime sleepiness, and a biomarker of neurodegeneration in patients with mild to severe OSAS. Clarifying the etiopathogenesis of cognitive impairment in OSAS remains crucial for defining optimal clinical interventions. Non-invasive ventilation (e.g., CPAP and BiPAP) is the gold-standard treatment for OSAS, effectively reducing respiratory events and improving sleep continuity and daytime sleepiness [22]. Sleepiness-related cognitive deficits may improve rapidly after treatment initiation, including short-term gains in verbal memory [23]. In contrast, impairments linked to hypoxia-related brain alterations may require longer recovery periods and may be only partially reversible.

To this end, we examined a cohort of Italian individuals admitted to the IRCCS Istituto Auxologico Italiano, San Giuseppe Hospital for a respiratory rehabilitation and weight-reduction program, who were enrolled in OSAS-related research projects during their stay. In addition to clinical data (i.e., the apnea–hypopnea index—AHI), measures of verbal memory (Selective Remind Test [24]) and problem-solving abilities (Tower of London [25]) were collected, as prior evidence indicates that these cognitive domains are among the most consistently affected in this clinical condition [12,13]. Indeed, these cognitive domains are thought to be particularly sensitive to OSAS-related mechanisms because they rely on fronto-hippocampal and fronto-subcortical networks that seem especially vulnerable to both neuroinflammation and sleep fragmentation [26].

Subjective sleepiness was assessed with the Epworth Sleepiness Scale [27] while serum neurofilament light chain (NfL) concentration was measured as an indicator of neuroaxonal damage. NfL is a structural component of the neuronal cytoskeleton that is released into the bloodstream following axonal injury or neurodegeneration [28]. Hypoxia-related neurodegenerative processes may promote cytoskeletal disruption and increased axonal membrane permeability, thereby facilitating the release of NfL into the peripheral circulation [29,30,31,32,33]. Previous evidence in OSAS indicates the presence of microstructural white-matter abnormalities and reduced axonal integrity [34,35] as well as elevated serum NfL levels [29,30,31], both of which correlate with disease severity and cognitive impairment. Accordingly, NfL may serve as a peripheral albeit non-specific marker of cumulative hypoxia-related neuronal and axonal injury, potentially helping to distinguish structurally mediated cognitive impairment from transient performance decrements related to excessive daytime sleepiness [36,37].

Despite growing interest in the link between OSAS and neurodegeneration, studies integrating neuropsychological assessment with biological markers sensitive to neuronal injury remain limited. By integrating clinical, cognitive and biomarker data, this study aims to provide novel insights into the mechanisms underlying cognitive dysfunction in OSAS and the sources of inter-individual variability in cognitive outcomes.

## 2. Materials and Methods

### 2.1. Participants

Participants included in this cross-sectional study were drawn from a pool of individuals with OSAS who participated in research activities during a three-week combined respiratory rehabilitation and weight-reduction program at IRCCS Istituto Auxologico Italiano, San Giuseppe Hospital between April 2021 and September 2025. According to institutional regulations, eligibility for this program requires the presence of obesity, which accounts for the high mean body mass index (BMI; kg/m^2^) observed in the sample.

OSAS diagnosis was established according to international guidelines, with inclusion requiring AHI greater than 5 events per hour of sleep. Following standard criteria [2,38], AHI values of 5 ≤ AHI < 15, 15 ≤ AHI < 30, and AHI ≥ 30 were used to classify mild, moderate, and severe OSAS, respectively. In line with rehabilitative purposes, all participants were affected by obesity (BMI kg/m^2^ ≥ 30).

All participants were ventilotherapy-naïve at hospital admission. Continuous positive airway pressure (CPAP) treatment was initiated during the rehabilitative stay as part of standard clinical care. Given evidence that even short-term exposure to CPAP may influence cognitive performance in OSAS [23] the number of days of CPAP use prior to neuropsychological assessment was extracted from clinical records and included as a covariate in subsequent analyses. Peripheral blood samples for NfL determination were collected in the morning under fasting conditions in close temporal proximity to the neuropsychological assessment, typically on the same day or within the surrounding days of testing.

Exclusion criteria included use of ventilatory therapy before the present rehabilitation program, a history of alcohol or substance abuse, psychiatric or neurological disorders, sensory deficits, or insufficient proficiency in the Italian language, in order to minimize potential confounding effects on cognitive performance and biomarker measures.

The study was conducted in accordance with the Declaration of Helsinki. Ethical approval was obtained for the original research projects from the institutional (IRCCS Istituo Auxologico Italiano, ID 2020_12_15_05) and local ethical board (Comitato Etico Territoriale Lombardia 5, ID 319/23) in which participants were enrolled during their rehabilitative stay at IRCCS Istituto Auxologico Italiano. Written informed consent for participation in the original studies and for the use of anonymized clinical and research data for research purposes was obtained from all participants.

### 2.2. Measures

Basic demographic information, including sex, age, and years of education, was collected for all participants. BMI was also recorded as an indicator of obesity, a clinical condition frequently associated with both OSAS and cognitive impairment [39,40].

The AHI was adopted as the primary measure of OSAS severity because it represents the internationally recognized and guideline-endorsed parameter for diagnosis and severity classification, allowing standardized categorization of disease severity and comparability with the existing clinical and research literature [39]. AHI values were derived from full-night polysomnography performed at hospital admission, prior to the initiation of ventilatory treatment.

Subjective daytime sleepiness was assessed using the Epworth Sleepiness Scale (ESS) [27] a widely used self-report questionnaire measuring perceived sleep propensity during daily activities and the likelihood of falling asleep in common situations. The ESS comprises eight items rated on a 4-point Likert scale (0 = “would never fall asleep” to 3 = “high chance of fall asleep”), with total scores obtained by summing item responses. A total score greater than 10 indicates excessive daytime sleepiness.

Neuropsychological performance was assessed by a trained neuropsychologist using standardized instruments.

Verbal memory and learning were evaluated with the Selective Reminding Test (SRT [24] a list-learning procedure across successive trials. Three indices are provided: (i) the long-term storage capacity (SRT-LTS) as measure of encoding efficiency, (ii) the consistency of long-term retrieval in successive trials (SRT-CLTR) as measure of verbal learning ability, and (iii) the delay recall of information (SRT-D) as measure of the persistence of learning in time. Higher scores are indicative of more efficient verbal memory and learning processes.

Executive functioning, specifically problem-solving and planning abilities, was assessed using the Tower of London test (ToL [25]). Both solution time (ToL-Time), reflecting processing speed, and the number of attempts required to reach the correct solution (ToL-Accuracy), reflecting performance accuracy, were recorded. Higher values reflect faster and more accurate performance.

NfL concentrations were determined from fasting morning peripheral blood samples. Blood was centrifuged at 3000× *g* for 10 min, and serum was aliquoted and stored at −80 °C until analysis. NfL levels were measured using the Simoa SR-X platform (Quanterix, Lexington, MA, USA) with a commercially available assay kit (catalog number 103400). All samples were analyzed in duplicate according to the manufacturer’s instructions. Inter-assay coefficients of variation were below 20%.

### 2.3. Statistical Analysis

Statistical analyses were performed using JASP software [41]. Analyses were conducted on complete cases, and no imputation procedures were applied. The associations between OSAS severity (AHI), daytime sleepiness (ESS total score), and neuronal injury (serum NfL) with cognitive performance were examined using a series of hierarchical multiple linear regression analyses, allowing the identification of the unique contribution of each predictor (or set of predictors) on the dependent variable at each step (i.e., block), over and above those considered in the previous steps.

Separate regression models were conducted for each cognitive outcome. Specifically, five models were estimated, with measures of verbal memory—long-term storage (SRT-LTS), verbal learning (SRT-CLTR), and delayed recall (SRT-D)—and problem-solving abilities—Tower of London completion time (ToL-Time) and accuracy (ToL-Accuracy)—entered as dependent variables. For each model, the performance score for the relevant cognitive measure was used as the outcome variable. In regression analyses, raw scores (i.e., not adjusted) were used to preserve variability, while age and education were explicitly controlled as covariates. Then, raw scores were adjusted according to guidelines and compared to normative data, in order to identify patients with a clinically relevant impairment in each cognitive outcome.

In each regression model, predictors were entered sequentially in blocks to evaluate their incremental contribution to explained variance. In all models, in Block 1, age, years of education, and BMI were included to account for their well-established effects on cognitive performance and to control for demographic and anthropometric variability. In Block 2, the number of days of continuous positive airway pressure (CPAP) treatment prior to neuropsychological assessment was entered, based on evidence indicating that even short-term exposure to ventilatory therapy may influence cognitive functioning. In Block 3, the primary predictors of interest were added simultaneously, including AHI, ESS total score, and serum NfL concentration. This hierarchical approach allowed assessment of the independent contributions of OSAS severity, subjective daytime sleepiness, and a biomarker of neuroaxonal injury beyond demographic, obesity-related, and treatment-related factors.

For each model, standardized regression coefficients (β), the total coefficient of determination (R^2^), the change in explained variance associated with the addition of Block 2 and Block 3 (ΔR^2^), and corresponding significance levels were reported. Partial correlation coefficients (|r|) were calculated as measures of effect size for individual predictors, reflecting the unique contribution of a predictor to the outcome after accounting for the variance explained by other covariates. Effects sizes were interpreted according to guidelines [42]: values of approximately |r| = 0.10 indicate small effects, |r| ≈ 0.30 indicate moderate effects, and |r| ≥ 0.50 indicates large effects. Assumptions of homoscedasticity and linearity were evaluated through visual inspection of residuals versus fitted values while normality of residuals was assessed using Q-Q plots and histograms. Casewise diagnostics were conducted to identify potential univariate outliers (studentized residuals exceeding ±3) and influential observations (Cook’s distance > 1; [43]). Multicollinearity was assessed using variance inflation factors (VIF), with values below 5 indicating the absence of problematic collinearity [44,45]. All analyses were conducted using a two-tailed significance threshold of *p* < 0.05.

## 3. Results

### 3.1. Participants

Seventy-two participants with OSAS were included in the study (36 females, 36 males; age: M = 56.22, SD = 10.11, range = 31–74 years; education: M = 12.01, SD = 4.37, range = 5–24 years; BMI: M = 45.42, SD = 7.13, range = 34.28–71.79 kg/m^2^). In line with diagnostic criteria, all participants had an AHI > 5. Most participants met the criteria for severe OSAS (*n* = 50), whereas nineteen presented moderate OSAS and three presented mild OSAS. Days since CPAP initiation (i.e., days of CPAP use prior to neuropsychological testing) ranged from 0 to 15 (M = 2.4, SD = 3.84). Descriptive statistics for the neuropsychological outcomes and the primary predictors (including sleepiness and biomarker measures) are reported in Table 1.

### 3.2. Model Diagnostic

For all five models, preliminary assumptions were met. Inspection of residuals-versus-predicted value plots indicated no evidence of non-linearity or heteroscedasticity, with residuals showing random dispersion around zero and constant variance across predicted values. The distribution of standardized residuals appeared approximately normal, as assessed through visual inspection of the residuals histograms and Q-Q plots. No univariate outliers (i.e., studentized residuals > ±3) or highly influential points (i.e., Cook’s distance > 1; [43]) were observed. Collinearity diagnostics indicated no multicollinearity concerns, with VIF values well below conventional thresholds [44,45] (see tables below)).

### 3.3. Verbal Memory

Regarding long term memory storage capacity, in Block 1, age, education, and BMI accounted for about 22% of variance observed in SRT-LTS scores (R^2^ = 0.22, F(3, 67) = 6.17, *p* < 0.001, R^2^ adj = 0.18). As illustrated in Table 2, age was a significant and moderate negative predictor (β = −0.36, *p* = 0.005, |r| = 0.34), whereas education and BMI were not significant contributors at this step (see Table 2). The addition of days of CPAP treatment in Block 2 produced a significant improvement in model fit (ΔR^2^ = 0.06, F(1, 66) = 5.84, *p* = 0.018, R^2^ adj = 0.24), increasing the explained variance to 28.0%. Days of CPAP emerged as a significant positive predictor of long-term memory storage capacity (β = 0.27, *p* = 0.016) indicating that greater exposure to CPAP treatment was associated with higher SRT-LTS scores (i.e., larger storage capacity) with a moderate effect (|r| = 0.26). In Block 3, the inclusion of the ESS score, AHI, and serum NfL concentrations did not significantly increase the explained variance (ΔR^2^ = 0.03, F(3, 63) = 0.90, *p* = 0.445, R^2^ adj = 0.23), and none of these variables were significant predictors after accounting for demographic, anthropometric, and treatment-related factors (all *p* > 0.149) (see Table 2). Overall, the final model explained 31.0% of the variance in SRT-LTS scores.

Regarding verbal learning, in *Block 1*, age, education, and BMI accounted for a significant proportion of variance in the SRT-CLTR scores (R^2^ = 0.26, F (3, 67) = 7.68, *p* < 0.001, R^2^ adj = 0.22). As reported in Table 3, age emerged as a significant moderate negative predictor (β = −0.34, *p* = 0.006, |*r*| = 0.33) while education showed a significant positive association (β = 0.25, *p* = 0.043) but with a smaller effect (|*r*| = 0.25) on verbal learning; conversely, BMI was not a significant predictor. The addition of days of CPAP treatment in *Block 2* produced a significant improvement in model fit (ΔR^2^ = 0.06, F(1, 66) = 5.57, *p* = 0.021, R^2^ adj = 0.27), with CPAP days positively and moderately predicting memory performance (β = 0.25, *p* = 0.021, |*r*| = 0.28). The model at this step explained 31.4% of the variance. However, in *Block 3*, the inclusion of the AHI, daytime sleepiness (ESS), and serum NfL concentrations did not significantly increase the explained variance (ΔR^2^ = 0.01, F(3, 63) = 0.20, *p* = 0.899, R^2^ adj = 0.25). None of these predictors were associated with the SRT-CLTR score after controlling for demographic and treatment-related variables (all *p* > 0.463).

Finally, relative to the ability to recall learnt verbal information, in *Block 1*, age, education, and BMI accounted for a significant amount of variance (R^2^ = 0.24, F(3, 67) = 7.16, *p* < 0.001, R^2^ adj = 0.21). As illustrated in Table 4, age was a significant and moderate negative predictor (β = −0.36, *p* = 0.004, |*r*| *=* 0.34), while neither education nor BMI contributed significantly at this step (see Table 4). The addition of days of CPAP treatment in *Block 2* significantly improved the model (ΔR^2^ = 0.07, F (1, 66) = 6.31, *p* = 0.014, R^2^ adj = 0.27), raising the explained variance to 31%. Days of CPAP emerged as a significant positive predictor (β = 0.27, *p* = 0.014), indicating that greater exposure to CPAP therapy was associated with better delayed recall performance with moderate effect (|*r*| *=* 0.30). Finally, in *Block 3*, the inclusion of ESS, AHI, and serum NfL concentration did not significantly increase the explained variance (ΔR^2^ = 0.018, F(3, 63) = 0.56, *p* = 0.646, R^2^ adj = 0.25). None of these predictors were significant after accounting for demographic, anthropometric, and treatment-related factors (all *p* > 0.28). The final model explained 32.7% of the variance in SRT-D scores.

Relative to all three memory outcomes, age consistently showed moderate negative associations, which is consistent with established age-related effects on memory performance, while CPAP treatment duration demonstrated small-to-moderate positive effects, suggesting that even short-term exposure to ventilatory therapy was associated with measurable improvements in memory-related performance. On the other hand, years of education showed a small positive effect only on verbal learning, but this association was attenuated after accounting for treatment-related factors. Crucially, body mass index did not exhibit meaningful associations with memory performance, suggesting that obesity in our sample did not significantly affect verbal memory performance. However, measures of OSAS severity (AHI), subjective daytime sleepiness (ESS), and markers of neuroaxonal injury (NfL) did not provide additional explanatory value when considering verbal memory.

### 3.4. Problem Solving

About the accuracy of problem-solving processes (see Table 5), in *Block 1*, age, years of education, and BMI did not account for a significant proportion of variance in ToL Accuracy scores (R^2^ = 0.02, F(3, 65) = 0.33, *p* = 0.803, R^2^ adj = −0.03). None of the predictors were significantly associated with performance (all *p* > 0.33), and partial correlations indicated negligible effect sizes (all |*r*| ≤ 0.12). The addition of days of CPAP treatment in *Block 2* did not significantly improve model fit (ΔR^2^ = 0.03, F(1, 64) = 2.071, *p* = 0.155) and the model remained non-significant overall (R^2^ = 0.05, R^2^ adj = −0.01), suggesting no significant contribution of the exposure to treatment on individual performance. Likewise, in *Block 3*, the inclusion of AHI, ESS, and serum NfL concentrations did not explain additional variance in ToL Accuracy score (ΔR^2^ = 0.02, F(3, 61) = 0.37, *p* = 0.772). The final model explained only 6.3% of the variance (R^2^ = 0.06), with a negative adjusted R^2^ (adj R^2^ = −0.04), indicating poor explanatory power. Thus, none of the clinical predictors were significantly associated with ToL Accuracy after controlling for demographic, anthropometric, and treatment-related variables (all *p* ≥ 0.315), as confirmed by the minimal effects sizes (all |r| ≤ 0.13).

Relative to the speed of problem-solving processes (see Table 6), in *Block 1*, age, years of education, and BMI did not account for a significant proportion of variance in ToL—Time (R^2^ = 0.01, F(3, 65) = 0.11, *p* = 0.953, R^2^ adj = −0.04). None of the predictors were significantly associated with performance (all *p* ≥ 0.625), and partial correlations indicated negligible effect sizes (|r| ≤ 0.06). The addition of days of CPAP treatment in *Block 2* resulted in a significant increase in explained variance (ΔR^2^ = 0.11, F(1, 64) = 8.31, *p* = 0.005), with the model accounting for 12.0% of the variance (R^2^ adj = 0.06). Days of CPAP treatment emerged as a significant positive predictor of problem-solving time (β = 0.36, *p* = 0.005), indicating that greater CPAP exposure was associated with lower task completion times, with a moderate effect size (|*r*| = 0.34). In *Block 3*, the inclusion of ESS, AHI, and serum NfL concentrations led to a non-significant increase in explained variance (ΔR^2^ = 0.09, F(3, 61) = 2.27, *p* = 0.09). The final model explained 20.8% of the variance (R^2^ adj = 0.12); however, none of the clinical predictors included in the last block reached statistical significance (all *p* > 0.07) (see Table 6).

For executive problem-solving outcomes, CPAP treatment duration was associated with a moderate effect on task completion time, whereas all other predictors, including OSAS severity (AHI), subjective daytime sleepiness (ESS), and serum NfL concentrations, showed negligible to small effects and did not reach statistical significance. Serum NfL exhibited a small-to-moderate trend-level association with ToL-Time, suggesting a potential but weak relationship with executive speed that did not meet conventional thresholds for statistical significance.

## 4. Discussion

This cross-sectional study aimed to contribute to the ongoing debate on the mechanisms underlying cognitive impairment in OSAS by examining the association between verbal memory and problem-solving abilities, OSAS severity, self-reported daytime sleepiness, and NfL serum concentration, as a possible marker of hypoxia-related neuroaxonal injury. In our sample, impairments in cognitive performance were observed primarily in the verbal learning and memory domain, with approximately 20% of participants scoring below the normative cutoff. In contrast, only a small proportion of patients showed clinically relevant deficits in problem-solving abilities, both in terms of processing speed (1.39%) and accuracy (4.17%). While a recent meta-analysis reports that up to 45% of patients with severe OSAS show cognitive impairment [46], this estimate refers to overall cognitive functioning rather than specific domains, for which pooled prevalence data remain scarce. Nonetheless, converging evidence indicates that verbal memory and executive functions are among the domains most consistently affected in OSAS, making the low rate of problem-solving impairment observed here partially divergent from previous findings [12,47]. This discrepancy may reflect, at least in part, differences in the operationalization of executive functions across studies. As noted by Buckets and colleagues [12] cognitive domains are often assessed using tests that capture distinct components or facets of the same domain; thus, the use of different measures may lead to partially divergent conclusions across studies. Most importantly, lack of variability in problem-solving abilities may have limited our ability to detect significant associations between this cognitive domain and the predictors examined in the present study.

Regarding the possible factors associated with cognitive performance in OSAS, clinical severity operationalized by the AHI did not predict cognitive performance in any of the domains examined, while remaining the gold standard for OSAS diagnosis and severity classification. This finding may appear counterintuitive. Nonetheless, it is consistent with previous evidence indicating that AHI is not reliably associated with performance in verbal memory or executive functions (e.g., Rey Auditory Verbal Learning Test, Stroop Test, Tower of London [48]). In fact, our findings extend the previous evidence by demonstrating that AHI may fail to predict cognitive functioning even in samples composed predominantly of patients with severe OSAS actively seeking medical care, rather than cohorts with milder cases. These results reinforce the notion that reliance on AHI alone may obscure clinically meaningful associations between OSAS and cognition; in contrast, alternative indices of nocturnal respiratory dysfunction may better capture pathophysiological mechanisms relevant to brain function. Measures reflecting hypoxic burden—such as nocturnal oxygen desaturation or average oxygen saturation—have been shown to be more closely associated with cognitive impairment and with structural brain changes in regions highly sensitive to oxygen supply, such as the amygdala and hippocampus [49]. Accordingly, we conducted additional sensitivity analyses in which the time spent with oxygen saturation below 90% (%T90) was entered as a predictor in place of AHI. This approach allowed us to isolate the contribution of cumulative hypoxic exposure while avoiding model overfitting due to the inclusion of highly correlated indices. Overall, models including %T90 showed a slight improvement in adjusted R^2^ compared to those including AHI but %T90 did not emerge as a significant predictor of cognitive performance. In contrast, age and duration of CPAP treatment were associated with all three memory indices, whereas problem-solving completion time was uniquely associated with treatment exposure (detailed results are reported in Supplementary Materials). These findings suggest that within our sample, lack of association between OSAS severity and cognition is robust across alternative operationalizations of sleep-disordered breathing severity. Future studies should integrate multiple severity metrics to more accurately elucidate the mechanisms underlying cognitive dysfunction in OSAS.

With respect to daytime sleepiness, our findings diverge from reports of negative associations between ESS scores and cognitive performance in patients with moderate-to-severe OSAS [50,51]. However, positive associations between ESS and cognitive performance in OSAS have been shown to be more pronounced in younger samples and when cognition is assessed using global screening instruments rather than domain-specific neuropsychological tests [51] or with no adequate consideration of possible confounding factors [52]. Indeed, our findings confirm the well-documented detrimental effects of aging [53] and protective role of cognitive reserve [54] in shaping cognitive outcomes since age emerged as a moderate and consistent predictor of all verbal memory indices, while years of education were selectively associated with better verbal learning. Furthermore, we may notice that subjective sleepiness measures showed limited convergence with objective indices [55], such as the Psychomotor Vigilance Task (PVT) or the Multiple Sleep Latency Test (MSLT), pointing to the importance for future studies that integrate both kinds of measures in OSAS research.

Likewise, serum NfL concentrations were not significantly associated with cognitive performance. Serum NfL showed only a small-to-moderate trend-level association with problem-solving speed, suggesting a potential but weak relationship that did not reach conventional thresholds for statistical significance. This result does not match preliminary evidence suggesting a significant association with cognitive impairments in adults with OSAS [56]. We hypothesized that biological interindividual differences, including greater neuronal resilience or cognitive reserve, as well as the multifactorial nature of cognitive dysfunction in adults with OSAS might blunt this association. Another plausible explanation for the lack of significant associations between serum NfL concentrations and cognitive performance in this study concerns the potentially non-linear and threshold-dependent nature of NfL release following axonal injury. Available evidence indicates that circulating NfL levels rise primarily when neuronal damage exceeds a certain severity, whereas milder or predominantly functional alterations may not result in measurable increases. In other words, NfL is thought to reflect cumulative and relatively advanced axonal injury. Within this framework, it is conceivable that, despite the predominance of severe OSAS in our sample, the extent of hypoxia-related neuronal damage did not reach a biological threshold sufficient to elicit detectable elevations in serum NfL. Also, cognitive deficits in OSAS may be related to altered brain functioning rather than to structural neural damage. This interpretation aligns with findings from neurodegenerative research, where associations between NfL, cognitive decline, and disease progression are typically stronger at higher concentration ranges and in more advanced pathological stages. Accordingly, the relatively low-to-moderate NfL levels observed in our cohort may have limited the sensitivity to detect robust relationships with cognitive outcomes.

An additional consideration is that blood NfL is a non-specific marker of neuroaxonal injury and its circulating concentrations can be influenced by several factors beyond hypoxemia-related neuronal damage. Age is a major determinant of NfL levels, which increase progressively across the adult lifespan even in the absence of overt neurological disease, likely to reflect age-related neuroaxonal vulnerability and cumulative subclinical pathology [57]. In the present study, the association between NfL concentrations and cognitive performance was examined over and above the well-established effect of age on cognition. In contrast, insufficient control for age-related variability in previous studies may partly account for inconsistencies with our findings. NfL concentrations may also be modulated by body composition, with obesity potentially affecting measured levels through blood volume-related dilution effects [58]. Moreover, NfL variability has been associated with renal function due to reduced clearance [59], systemic inflammation and vascular–metabolic comorbidities [60]. Given that our sample consisted of severely obese inpatients, who are likely to present a higher burden of cardiovascular, metabolic disease and possible renal dysfunction, these factors may have contributed additional variability in NfL concentrations and obscured subtle associations with cognitive performance. Overall, our findings suggest that although NfL holds promise as a biomarker of neuronal injury [31,36], its utility as a hypoxia-related indicator of cognitive impairment in adult OSAS populations should be probed more deeply, systematically accounting for comorbidities and relevant biological covariates.

Indeed, in our linear regression models, only exposure to continuous positive airway pressure (CPAP) emerged as a significant clinical predictor of all verbal memory indices and of problem-solving speed. Although the present design does not allow causal inference, this pattern is consistent with a detrimental effect of untreated OSAS on cognitive functioning and suggests that early treatment exposure may be associated with measurable cognitive benefits, like we previously observed [23]. The mechanisms underlying these relationships remain unclear but, considering the short CPAP exposure, better cognitive functioning might be more likely related to acute sleepiness recovery than reversibility of hypoxia-related neural alterations. Furthermore, CPAP exposure in the present study was operationalized as the number of days since first CPAP adaptation, irrespective of effective nightly use. This approach captures differences in the timing of treatment initiation within the rehabilitative program rather than adherence per se. However, this measure does not account for interindividual variability in actual CPAP usage, which may be influenced by motivation, tolerance, and compliance. Therefore, future studies should incorporate objective indices of effective CPAP use (e.g., average hours of use per night and adherence rates), to disentangle treatment exposure from adherence-related factors and to characterize more accurately the relationship between CPAP therapy and cognitive outcomes. Furthermore, patients with longer CPAP exposure were also those who had been enrolled in the rehabilitative program for a longer duration. As part of this program, participants engaged in structured physical activity, which has been consistently associated with improvements in cognitive function in both individuals with OSAS [61] and obesity [62]. Therefore, it cannot be excluded that components of the rehabilitation program other than CPAP contributed to the observed association with cognitive outcomes.

Additionally, methodological limitations may partly account for the absence of the expected associations between cognitive performance, daytime sleepiness, and serum NfL concentrations. The cross-sectional, within-subject design—lacking both a control group and longitudinal cognitive follow-up—precludes causal inference. Additional constraints include the absence of complementary hypoxemia indices, a limited assessment of cognitive domains, and the short and heterogeneous duration of CPAP exposure prior to evaluation. Although the observation-to-predictor ratio met the lower bound of commonly cited recommendations [63], the modest sample size relative to the number of predictors may have increased the risk of overfitting, warranting a cautious and exploratory interpretation of the regression findings pending replication in larger samples. Further limitations concern sample characteristics. The study exclusively included inpatients enrolled in a respiratory rehabilitation and weight reduction program, resulting in a sample characterized by severe obesity. This feature, together with the predominance of patients with severe OSAS, may have introduced ceiling effects and reduced variability in both BMI and disease severity, potentially limiting the detection of associations with cognitive outcomes and restricting the generalizability of the findings to outpatient OSAS populations. Moreover, cardiovascular and metabolic comorbidities—closely linked to both obesity and OSAS as well as cognitive impairment [64,65,66,67]—were not fully controlled and may have acted as residual confounders. Overall, design-related constraints, sample characteristics, and potential selection bias may have reduced statistical power and hindered the identification of subtle relationships among cognition, sleepiness, and NfL. In addition, effect sizes across all regression models were modest (i.e., in the small-to-moderate range), which constrains the clinical interpretability and translational relevance of the observed associations. Future studies should adopt longitudinal designs in larger and more heterogeneous samples, including individuals with a wider range of BMI and OSAS severity, and incorporate broader cognitive batteries, objective sleepiness measures, multiple hypoxemia indices, and biomarkers assessed before and after sustained CPAP exposure. Despite these limitations, the present study provides preliminary evidence that, within a clinically severe OSAS sample, variability in cognitive performance is more strongly related to stable demographic factors and early treatment exposure than to OSAS severity, subjective sleepiness, or peripheral markers of neuroaxonal injury. These findings highlight the need for multidimensional severity assessment and longitudinal designs to clarify the complex pathways linking OSAS, brain health, and cognitive outcomes.

## Figures and Tables

**Table 1:** Means, standard deviations (SD) and range values relative to dependent variables (i.e., neuropsychological tests raw scores) and primary predictors. For neuropsychological tests, % of patients with impaired performance compared to normative data is reported. About ESS score, % of patients with clinically relevant daytime sleepiness (score ≥ 10) are shown.

	N	Mean	SD	Min.	Max.	% Impaired
**SRT-LTS**	72	35.90	16.05	5	72	23.61
**SRT-CLTR**	72	27.94	15.73	0	72	18.06
**SRT-D**	72	7.25	2.95	2	12	27.78
**ToL-Time**	70 *	28.52	4.01	19	36	1.39
**ToL-Accuracy**	70 *	29.98	3.10	22	36	4.17
**AHI**	72	52.93	30.82	5.50	138.60	-
**NfL (pg/mL)**	72	13.84	11.17	3.65	84.77	-
**ESS**	72	7.49	5.27	0	24	29.17

*Note*: SRT-LTS: Selective Reminding Test—Long Term Storage; SRT-CLTR: Selective Reminding Test—Consistency of Long-Term Retrieval; SRT-D: Selective Reminding Test—Delay recall; ToL-Time: Tower of London—Time; ToL-Accuracy: Tower of London—Accuracy as measure of strategic reasoning, problem-solving, and mental planning. * Two participants had missing ToL data; therefore, analyses involving ToL outcomes (ToL-Time and ToL-Accuracy) were performed on complete cases (*n* = 70).

**Table 2:** Results of the hierarchical multiple linear regression analysis investigating the possible predictors of long-term verbal memory storage capacity (i.e., SRT-LTS scores). R^2^, adjusted R^2^, and F-statistics are reported for each model. Standardized β coefficients, *t*-values, *p*-values, partial *r* correlation and 95% CI, coefficients and VIF values are reported for each predictor in each model. Statistical significance is in bold.

		β	*t*	*p*	Partial *r* [95% CI]		VIF
*Block 1*	R^2^ = 0.22, R^2^ adj = 0.18 (F_3,67_ = 6.17, *p* = <0.001)						
	(Constant)		2.40	0.016			
	**Age**	−0.36	−2.93	**0.005**	−0.34	[−0.54, −0.1]	1.27
	**Education—years**	0.15	1.20	0.233	0.15	[−0.1, 0.37]	1.27
	**BMI (kg/m^2^)**	0.12	1.13	0.262	0.14	[−0.11, 0.37]	1.04
*Block 2*	R^2^ = 0.28, R^2^ adj = 0.24 (F_4.66_ = 6.42, *p* = <0.001)						
	(Constant)		3.08	0.003			
	**Age**	−0.44	−3.58	<**0.001**	−0.40	[−0.59, −0.18]	1.38
	**Education—years**	0.12	0.98	0.333	0.12	[−0.13,0.35]	1.29
	**BMI (kg/m^2^)**	0.07	0.62	0.537	0.08	[−0.17, 0.31]	1.09
	**Days of CPAP**	0.27	2.42	**0.018**	0.29	[0.05, 0.49]	1.12
*Block 3*	R^2^ = 0.31, R^2^ adj = 0.23 (F_7.63_ = 4.04, *p* = 0.001)						
	(Constant)		2.93	0.005			
	**Age**	−0.39	−3.09	**0.003**	−0.36	[−0.56, −0.13]	1.48
	**Education—years**	0.12	0.95	0.346	0.12	[−0.13, 0.35]	1.43
	**BMI (kg/m^2^)**	−0.00	−0.01	0.988	0.00	[−0.24, 0.24]	1.29
	**Days of CPAP**	0.29	2.55	**0.013**	0.31	[0.07, 0.51]	1.15
	**ESS**	0.17	1.46	0.149	0.18	[−0.06, 0.41]	1.20
	**AHI**	0.03	0.26	0.797	0.03	[−0.21, 0.27]	1.20
	**NfL (pg/mL)**	−0.08	−0.66	0.512	−0.08	[−0.32, 0.16]	1.24

*Note.* BMI: body mass index; AHI: apnea-hypopnea index; Ess—Total: Epworth Sleepiness Scale total score; NfL: light chain neurofilament.

**Table 3:** Results of the hierarchical multiple linear regression analysis investigating the possible predictors of consistency of verbal learning (i.e., SRT-CLTR scores). R^2^, adjusted R^2^, and F-statistics are reported for each model. Standardized β coefficients, *t*-values, *p*-values, partial *r* correlation and 95% CI, coefficients and VIF values are reported for each predictor in each model. Statistical significance is in bold.

		β	*t*	*p*	Partial *r* [95% CI]		VIF
*Block 1*	R^2^ = 0.26, R^2^ adj = 0.22 (F_3,67_ = 7.68, *p* = <0.001)						
	(Constant)		2.07	0.043			
	**Age**	−0.34	−2.84	**0.006**	−0.33	[−0.53, −0.09]	1.27
	**Education—years**	0.25	2.07	**0.043**	0.25	[0.003, 0.46]	1.27
	**BMI (kg/m^2^)**	0.09	0.80	0.428	0.10	[−0.15, 0.33]	1.04
*Block 2*	R^2^ = 0.31, R^2^ adj = 0.27 (F_4.66_ = 7.54, *p* = <0.001)						
	(Constant)		2.65	0.010			
	**Age**	−0.42	−3.47	<**0.001**	−0.39	[−0.58, −0.17]	1.38
	**Education—years**	0.22	1.87	0.067	0.22	[−0.02, 0.44]	1.29
	**BMI (kg/m^2^)**	0.03	0.29	0.769	0.04	[−0.21, 0.28]	1.09
	**Days of CPAP**	0.25	2.36	**0.021**	0.28	[0.04, 0.49]	1.12
*Block 3*	R^2^ = 0.32, R^2^ adj = 0.24 (F_7.63_ = 4.24, *p* = <0.001)						
	(Constant)		2.52	0.014			
	**Age**	−0.40	−3.18	**0.002**	−0.37	[−0.56, −0.14]	1.48
	**Education—years**	0.21	1.71	0.092	0.21	[−0.03, 0.43]	1.43
	**BMI (kg/m^2^)**	0.01	0.07	0.945	0.01	[−0.23, 0.25]	1.29
	**Days of CPAP**	0.26	2.36	**0.022**	0.29	[0.05, 0.49]	1.15
	**ESS**	0.08	0.74	0.463	0.09	[−0.15, 0.33]	1.20
	**AHI**	−0.02	−0.20	0.838	−0.03	[−0.27, 0.22]	1.20
	**NfL (pg/mL)**	−0.03	−0.27	0.789	−0.03	[−0.27, 0.21]	1.24

*Note.* BMI: body mass index; AHI: apnea-hypopnea index; Ess—Total: Epworth Sleepiness Scale total score; NfL: light chain neurofilament.

**Table 4:** Results of the hierarchical multiple linear regression analysis investigating the possible predictors of delayed recall of verbal information (i.e., SRT-D). R^2^, adjusted R^2^, and F-statistics are reported for each model. Standardized β coefficients, *t*-values, *p*-values, partial *r* correlation and 95% CI, correlation coefficients and VIF values are reported for each predictor in each model. Statistical significance is in bold.

		β	*t*	*p*	Partial *r* [95% CI]		VIF
*Block 1*	R^2^ = 0.24, R^2^ adj = 0.21 (F_3,67_ = 7.16, *p* = <0.001)						
	(Constant)		3.16	0.002			
	**Age**	−0.35	−2.96	**0.004**	−0.34	[−0.54, −0.11]	1.27
	**Education—years**	0.22	1.83	0.072	0.22	[−0.03, 0.44]	1.27
	**BMI (kg/m^2^)**	0.00	0.02	0.983	0.00	[−0.24, 0.24]	1.04
*Block 2*	R^2^ = 0.31, R^2^ adj = 0.27 (F_4.66_ = 7.37, *p* = <0.001)						
	(Constant)		3.80	<0.001			
	**Age**	−0.44	−3.66	<**0.001**	−0.41	[−0.59, −0.19]	1.38
	**Education—years**	0.19	1.62	0.111	0.20	[−0.05, 0.42]	1.29
	**BMI (kg/m^2^)**	−0.06	−0.52	0.604	−0.06	[−0.3, 0.18]	1.09
	**Days of CPAP**	0.27	2.51	**0.014**	0.30	[0.06, 0.5]	1.12
*Block 3*	R^2^ = 0.33, R^2^ adj = 0.25 (F_7.63_ = 4.37, *p* = <0.001)						
	(Constant)		3.53	<0.001			
	**Age**	−0.44	−3.50	<**0.001**	−0.40	[−0.59, −0.18]	1.48
	**Education—years**	0.20	1.63	0.109	0.20	[−0.04, 0.42]	1.43
	**BMI (kg/m^2^)**	−0.06	−0.53	0.599	−0.07	[−0.3, 0.18]	1.29
	**Days of CPAP**	0.29	2.60	**0.011**	0.31	[0.08, 0.52]	1.15
	**ESS**	0.12	1.09	0.280	0.14	[−0.11, 0.37]	1.20
	**AHI**	−0.07	−0.61	0.542	−0.08	[−0.31, 0.17]	1.20
	**NfL (pg/mL)**	0.04	0.39	0.695	0.05	[−0.2, 0.29]	1.24

*Note.* BMI: body mass index; AHI: apnea-hypopnea index; Ess—Total: Epworth Sleepiness Scale total score; NfL: light chain neurofilament.

**Table 5:** Results of the hierarchical multiple linear regression analysis investigating the possible predictors of accuracy in problem solving abilities (i.e., ToL—Accuracy). R^2^, adjusted R^2^, and F-statistics are reported for each model. Standardized β coefficients, *t*-values, *p*-values, partial *r* correlation and 95% CI, coefficients and VIF values are reported for each predictor in each model.

		β	*t*	*p*	Partial *r* [%95CI]		VIF
*Block 1*	R^2^ = 0.01 R^2^ adj = −0.03 (F_3,65_ = 0.33, *p* = 0.803)						
	(Constant)		7.54	<0.001			
	**Age**	−0.03	−0.22	0.828	−0.03	[−0.27, 0.22]	1.26
	**Education—years**	−0.01	−0.05	0.962	−0.01	[−0.25, 0.24]	1.27
	**BMI (kg/m^2^)**	−0.12	−0.98	0.330	−0.12	[−0.35, 0.12]	1.04
*Block 2*	R^2^ = 0.05 R^2^ adj = −0.01 (F_4.64_ = 0.77, *p* = 0.549)						
	(Constant)		7.72	<0.001			
	**Age**	−0.09	−0.62	0.538	−0.08	[−0.31, 0.17]	1.37
	**Education—years**	−0.03	−0.20	0.844	−0.03	[−0.27, 0.22]	1.28
	**BMI (kg/m^2^)**	−0.16	−1.28	0.206	−0.16	[−0.39, 0.09]	1.09
	**Days of CPAP**	0.19	1.43	0.155	0.18	[−0.07, 0.4]	1.12
*Block 3*	R^2^ = 0.06 R^2^ adj = −0.04 (F_7.61_ = 0.59, *p* = 0.764)						
	(Constant)		7.21	<0.001			
	**Age**	−0.07	−0.49	0.628	−0.06	[−0.3, 0.18]	1.47
	**Education—years**	−0.01	−0.05	0.961	−0.01	[−0.25, 0.24]	1.42
	**BMI (kg/m^2^)**	−0.18	−1.28	0.204	−0.16	[−0.39, 0.08]	1.31
	**Days of CPAP**	0.19	1.40	0.166	0.18	[−0.07, 0.4]	1.16
	**ESS**	−0.06	−0.44	0.659	−0.06	[−0.29, 0.19]	1.21
	**AHI**	0.14	1.01	0.315	0.13	[−0.12, 0.36]	1.20
	**NfL (pg/mL)**	0.00	0.03	0.978	0.00	[−0.24, 0.25]	1.23

*Note.* BMI: body mass index; AHI: apnea-hypopnea index; Ess—Total: Epworth Sleepiness Scale total score; NfL: light chain neurofilament.

**Table 6:** Results of the hierarchical multiple linear regression analysis investigating the possible predictors of speed of problem-solving processes (i.e., ToL Time). R^2^, adjusted R^2^, and F-statistics are reported for each model. Standardized β coefficients, *t*-values, *p*-values, partial *r* correlation and 95% CI, coefficients and VIF values are reported for each predictor in each model. Statistical significance is in bold.

		β	*t*	*p*	Partial *r* [95% CI]		VIF	
*Block 1*	R^2^ = 0.00 R^2^ adj = −0.04 (F_3,65_ = 0.11, *p* = 0.953)							
	(Constant)		5.21	<0.001				
	**Age**	0.01	0.06	0.948	0.01	[−0.23, 0.25]	1.26	
	**Education—years**	0.03	0.22	0.829	0.03	[−0.22, 0.27]	1.27	
	**BMI (kg/m^2^)**	−0.06	−0.49	0.625	−0.06	[−0.3, 0.18]	1.04	
*Block 2*	R^2^ = 0.12 R^2^ adj = 0.06 (F_4.64_ = 2.17, *p* = 0.082)							
	(Constant)		6.03	<0.001				
	**Age**	−0.10	−0.75	0.455	−0.09	[−0.33, 0.15]	1.37	
	**Education—years**	−0.01	−0.07	0.942	−0.01	[−0.25, 0.23]	1.28	
	**BMI (kg/m^2^)**	−0.14	−1.13	0.261	−0.14	[−0.37, 0.11]	1.09	
	**Days of CPAP**	0.36	2.88	0.005	0.34	[0.11, 0.54]	1.12	
*Block 3*	R^2^ = 0.21 R^2^ adj = 0.12 (F_7.61_ = 2.29, *p* = 0.039)							
	(Constant)		6.33	<0.001				
	**Age**	−0.07	−0.50	0.618	−0.06	[−0.3, 0.18]	1.47	
	**Education—years**	−0.07	−0.49	0.626	−0.06	[−0.3, 0.18]	1.42	
	**BMI (kg/m^2^)**	−0.17	−1.32	0.193	−0.17	[−0.39, 0.08]	1.31	
	**Days of CPAP**	0.32	2.60	0.012	0.32	[0.08, 0.52]	1.16	
	**ESS**	−0.17	−1.35	0.183	−0.17	[−0.4, 0.08]	1.21	
	**AHI**	0.13	1.05	0.296	0.13	[−0.11, 0.36]	1.20	
	**NfL (pg/mL)**	−0.23	−1.84	0.070	−0.23	[−0.45, 0.01]	1.23	

*Note.* BMI: body mass index; AHI: apnea-hypopnea index; Ess—Total: Epworth Sleepiness Scale total score; NfL: light chain neurofilaments.

## Data Availability

Research data are available upon reasonable requests at https://doi.org/10.5281/zenodo.18016286.

## Supplementary Materials

The following supporting information can be downloaded at: https://www.mdpi.com/article/10.3390/jcm15041588/s1, Tables S1: Results of the hierarchical multiple linear regression analysis investigating the possible predictors of long-term verbal memory storage capacity (i.e., SRT-LTS scores); Table S2: Results of the hierarchical multiple linear regression analysis investigating the possible predictors of verbal learning (i.e., SRT-CLTR scores); Table S3: Results of the hierarchical multiple linear regression analysis investigating the possible predictors of verbal delay recall (i.e., SRT-D); Table S4: Results of the hierarchical multiple linear regression analysis investigating the possible predictors of problem-solving speed (i.e., ToL Time); Table S5: Results of the hierarchical multiple linear regression analysis investigating the possible predictors of problem-solving accuracy (i.e., ToL Accuracy).

## Abbreviations

The following abbreviations are used in this manuscript:

OSAS	Obstructive Sleep Apnea Syndrome
AHI	Apnea–hypopnea Index
SRT	Selective Reminding Test
ToL	Tower of London
ESS	Epworth Sleepiness Scale
NfL	Neurofilament light chain
