# Supplementary material for: CPAP Treatment Exposure, but Not Daytime Sleepiness or Neurofilament Light Chain, Is Associated with Cognitive Performance in Obstructive Sleep Apnea
Source: J Clin Med. 2026 Feb 18;15(4):1588. doi: 10.3390/jcm15041588 (PMC12942159; doi:10.3390/jcm15041588)
Supplement: Supplementary file 1 [file jcm-15-01588-s001.zip › jcm-4086995-supplementary.pdf]

CPAP treatment exposure, but not daytime sleepiness or neurofilament light chain, is associated with cognitive performance in obstructive sleep apnea.

Supplementary Materials

Sofia Tagini, Stefania Cattaldo, Federica Scarpina, Erica Sabattini, Giulia Chirchio, Elisa Prina, Paolo Piterà, Clara Paschino, Riccardo Cremascoli, Mirna Solange Barrio Lower Daniele, Mauro Cornacchia, Theodore Tsaras, Amelia Brunani, Massimo Scacchi, Paolo Fanari, Alessandro Mauro, Lorenzo Priano

Sensitivity analyses

As a sensitivity analysis, all linear regression models were re-estimated by replacing the Apnea–Hypopnea Index (AHI) with the percentage of total sleep time spent with oxygen saturation below 90% (%T90), an index reflecting cumulative nocturnal hypoxic burden. This approach was adopted to evaluate whether hypoxemia-related severity, rather than respiratory event frequency, was more strongly associated with cognitive outcomes, while avoiding overfitting and collinearity arising from the simultaneous inclusion of multiple OSAS severity indices. Compared to the primary models, those including %T90 showed a slight improvement in adjusted R^2; however, the overall pattern of results remained unchanged, and %T90 did not emerge as a significant predictor in any model. These findings indicate that the absence of significant associations between OSAS severity and cognitive performance was robust across alternative operationalizations of sleep-disordered breathing severity. Detailed statistics for each outcome are reported below.

Table S1. Results of the hierarchical multiple linear regression analysis investigating the possible predictors of long-term verbal memory storage capacity (i.e., SRT-LTS scores). R^2, adjusted R^2, and F-statistics are reported for each model. Standardized β coefficients, *t*-values, *p*-values, partial *r* correlation and 95% CI, coefficients and VIF values are reported for each predictor in each model. Statistical significance is in bold.

		β	*t*	*p*	Partial *r*	[%95CI]	VIF
**Block 1**	R2 = 0.23 R2 adj = 0.20 ($F_{3,67}$ = 6.57, p = < .001)						
	(Constant)		2.51	0.015			
	Age	-0.37	-3.04	**0.003**	-0.35	[-0.55, -0.12]	1.28
	Education - years	0.15	1.26	0.212	0.15	[-0.09, 0.38]	1.27
	BMI (kg/m2)	0.11	0.96	0.341	0.12	[-0.13, 0.35]	1.06
**Block 2**	R2 = 0.28 R2 adj = 0.24 ($F_{4,66}$ = 6.38, p = < .001)						
	(Constant)		2.86	0.006			
	Age	-0.43	-3.53	**< .001**	-0.40	[-0.59, -0.18]	1.35
	Education - years	0.13	1.07	0.287	0.13	[-0.11, 0.36]	1.29

	BMI (kg/m2)	0.09	0.79	0.430	0.10 [-0.15, 0.33]	1.07
	Days of CPAP	0.23	2.17	**0.034**	0.26 [0.02, 0.47]	1.06
**Block 3**	R2 = 0.32 R2 adj = 0.25 ($F_{7,63}$ = 4.17, p = < .001)					
	(Constant)		2.63	0.011		
	Age	-0.42	-3.30	**0.002**	-0.39 [-0.58, -0.16]	1.44
	Education - years	0.15	1.17	0.246	0.15 [-0.1, 0.38]	1.43
	BMI (kg/m2)	0.03	0.21	0.835	0.03 [-0.22, 0.27]	1.26
	Days of CPAP	0.24	2.22	**0.030**	0.27 [0.03, 0.48]	1.08
	ESS	0.15	1.29	0.200	0.16 [-0.08, 0.39]	1.20
	%T90	0.14	1.24	0.220	0.16 [-0.09, 0.38]	1.08
	NfL (pg/mL)	-0.04	-0.33	0.742	-0.04 [-0.28, 0.2]	1.31

Note. BMI: body mass index; %T90: percentage of total sleep time spent with oxygen saturation below 90%; Ess - Total: Epworth Sleepiness Scale total score; NfL: light chain neurofilament.

Table S2. Results of the hierarchical multiple linear regression analysis investigating the possible predictors of verbal learning (i.e., SRT-CLTR scores). R^2, adjusted R^2, and F-statistics are reported for each model. Standardized β coefficients, *t*-values, *p*-values, partial *r* correlation and 95% CI, coefficients and VIF values are reported for each predictor in each model. Statistical significance is in bold.

		**β**	***t***	***p***	**Partial *r* [%95CI]**	**VIF**
**Block 1**	R2 = 0.27 R2 adj = 0.23 ($F_{3,67}$ = 7.90, p = < .001)					
	(Constant)		1.96	0.055		
	Age	-0.34	-2.83	**0.006**	-0.33 [-0.53, -0.1]	1.28
	Education - years	0.26	2.13	0.037	0.26 [0.01, 0.47]	1.27
	BMI (kg/m2)	0.09	0.79	0.431	0.10 [-0.15, 0.33]	1.06
**Block 2**	R2 = 0.33 R2 adj = 0.29 ($F_{4,66}$ = 7.93, p = < .001)					
	(Constant)		2.36	0.021		
	Age	-0.41	-3.43	**0.001**	-0.39 [-0.58, -0.17]	1.35
	Education - years	0.23	1.96	0.055	0.24 [-0.01, 0.45]	1.29
	BMI (kg/m2)	0.06	0.60	0.548	0.08 [-0.17, 0.31]	1.07
	Days of CPAP	0.26	2.48	**0.016**	0.30 [0.06, 0.5]	1.06
**Block 3**	R2 = 0.34 R2 adj = 0.27 ($F_{7,63}$ = 4.56, p = < .001)					
	(Constant)		2.07	0.042		

Age	-0.41	-3.31	**0.002**	-0.39	[-0.58, -0.16]	1.44
Education - years	0.25	2.01	**0.049**	0.25	[0.01, 0.46]	1.43
BMI (kg/m2)	0.05	0.40	0.689	0.05	[-0.19, 0.29]	1.26
Days of CPAP	0.27	2.45	**0.017**	0.30	[0.06, 0.51]	1.08
ESS	0.05	0.44	0.662	0.06	[-0.19, 0.29]	1.20
%T90	0.10	0.96	0.343	0.12	[-0.12, 0.35]	1.08
NfL (pg/mL)	0.02	0.14	0.885	0.02	[-0.22, 0.26]	1.31

Note. BMI: body mass index; %T90: percentage of total sleep time spent with oxygen saturation below 90%; Ess - Total: Epworth Sleepiness Scale total score; NfL: light chain neurofilament.

Table S3. Results of the hierarchical multiple linear regression analysis investigating the possible predictors of verbal delay recall (i.e., SRT-D). R^2, adjusted R^2, and F-statistics are reported for each model. Standardized β coefficients, *t*-values, *p*-values, partial *r* correlation and 95% CI, coefficients and VIF values are reported for each predictor in each model. Statistical significance is in bold.

		**β**	***t***	***p***	**Partial *r* [%95CI]**		**VIF**
**Block 1**	R2 = 0.25 R2 adj = 0.21 ($F_{3,67}$ = 7.17, p = < .001)						
	(Constant)		3.18	0.002			
	Age	-0.37	-3.01	0.004	-0.35	[-0.55, -0.12]	1.28
	Education - years	0.21	1.77	0.081	0.22	[-0.03, 0.43]	1.27
	BMI (kg/m2)	-0.02	-0.17	0.867	-0.02	[-0.26, 0.22]	1.06
**Block 2**	R2 = 0.31 R2 adj = 0.27 ($F_{4,66}$ = 7.24, p = < .001)						
	(Constant)		3.61	< 0.001			
	Age	-0.43	-3.59	**< .001**	-0.41	[-0.59, -0.19]	1.35
	Education - years	0.19	1.59	0.117	0.20	[-0.05, 0.42]	1.29
	BMI (kg/m2)	-0.04	-0.39	0.702	-0.05	[-0.29, 0.2]	1.07
	Days of CPAP	0.26	2.42	**0.018**	0.29	[0.05, 0.5]	1.06
**Block 3**	R2 = 0.33 R2 adj = 0.25 ($F_{7,63}$ = 4.24, p = < .001)						
	(Constant)		3.19	.002			
	Age	-0.43	-3.41	**0.001**	-0.40	[-0.59, -0.17]	1.44
	Education - years	0.22	1.75	0.084	0.22	[-0.02, 0.44]	1.43
	BMI (kg/m2)	-0.06	-0.52	0.603	-0.07	[-0.3, 0.18]	1.26
	Days of CPAP	0.28	2.53	**0.014**	0.31	[0.07, 0.51]	1.08

		β	t	p	Partial r	[%95CI]	VIF
	ESS	0.11	0.95	0.348	0.12	[-0.13, 0.35]	1.20
	%T90	0.04	0.39	0.700	0.05	[-0.19, 0.29]	1.08
	NfL (pg/mL)	0.07	0.54	0.593	0.07	[-0.18, 0.31]	1.31

Note. BMI: body mass index; %T90: percentage of total sleep time spent with oxygen saturation below 90%; Ess - Total: Epworth Sleepiness Scale total score; NfL: light chain neurofilament.

Table S4. Results of the hierarchical multiple linear regression analysis investigating the possible predictors of problem-solving speed (i.e., ToL Time). R^2, adjusted R^2, and F-statistics are reported for each model. Standardized β coefficients, *t*-values, *p*-values, partial *r* correlation and 95% CI, coefficients and VIF values are reported for each predictor in each model. Statistical significance is in bold.

		**β**	***t***	***p***	**Partial *r***	**[%95CI]**	**VIF**
**Block 1**	R2 = 0.01 R2 adj = -0.04 ($F_{3,65}$ = 0.15, p = 0.930)						
	(Constant)		5.06	<0.001			
	Age	-0.00	-0.02	0.983	0.00	[-0.24, 0.24]	1.27
	Education - years	0.03	0.20	0.842	-0.07	[-0.31, 0.17]	1.27
	BMI (kg/m2)	-0.07	-0.58	0.562	0.03	[-0.22, 0.27]	1.06
**Block 2**	R2 = 0.13 R2 adj = 0.08 ($F_{4,64}$ = 2.39, p = 0.060)						
	(Constant)		5.75	<0.001			
	Age	-0.10	-0.72	0.472	-0.09	[-0.33, 0.15]	1.35
	Education - years	-0.01	-0.07	0.945	-0.11	[-0.34, 0.13]	1.28
	BMI (kg/m2)	-0.11	-0.88	0.381	-0.01	[-0.25, 0.23]	1.07
	Days of CPAP	0.37	3.01	**0.004**	0.36	[0.13, 0.55]	1.06
**Block 3**	R2 = 0.20 R2 adj = 0.11 ($F_{7,61}$ = 2.17, p = 0.050)						
	(Constant)		6.14	<0.001			
	Age	-0.08	-0.60	0.547	-0.08	[-0.32, 0.17]	1.44
	Education - years	-0.09	-0.68	0.501	-0.11	[-0.34, 0.14]	1.42
	BMI (kg/m2)	-0.11	-0.85	0.399	-0.09	[-0.32, 0.16]	1.28
	Days of CPAP	0.32	2.63	**0.011**	0.32	[0.09, 0.52]	1.09
	ESS	-0.15	-1.18	0.245	-0.15	[-0.38, 0.09]	1.23
	%T90	-0.03	-0.25	0.802	-0.03	[-0.27, 0.21]	1.08
	NfL (pg/mL)	-0.24	-1.80	0.077	-0.23	[-0.45, 0.01]	1.30

Note. BMI: body mass index; %T90: percentage of total sleep time spent with oxygen saturation below 90%; Ess - Total: Epworth Sleepiness Scale total score; NfL: light chain neurofilament.

Table S5. Results of the hierarchical multiple linear regression analysis investigating the possible predictors of problem-solving accuracy (i.e., ToL Accuracy). R^2, adjusted R^2, and F-statistics are reported for each model. Standardized β coefficients, *t*-values, *p*-values, partial *r* correlation and 95% CI, coefficients and VIF values are reported for each predictor in each model.

		β	*t*	*p*	Partial *r*	[%95CI]	VIF
**Block 1**	R2 = 0.01 R2 adj = -0.04 ($F_{3,65}$ = 0.27, p = 0.850)						
	(Constant)		7.12	<0.001			
	Age	-0.03	-0.22	0.829	-0.03	[-0.27, 0.22]	1.27
	Education - years	0.00	0.01	0.993	0.00	[-0.24, 0.24]	1.27
	BMI (kg/m2)	-0.11	-0.86	0.391	-0.11	[-0.34, 0.14]	1.06
**Block 2**	R2 = 0.05 R2 adj = -0.01 ($F_{4,64}$ = 0.85, p = 0.500)						
	(Constant)		7.37	<0.001			
	Age	-0.08	-0.59	0.559	-0.07	[-0.31, 0.17]	1.35
	Education - years	-0.02	-0.14	0.888	-0.02	[-0.26, 0.23]	1.28
	BMI (kg/m2)	-0.13	-1.01	0.315	-0.13	[-0.36, 0.12]	1.07
	Days of CPAP	0.20	1.61	0.113	0.20	[-0.04, 0.42]	1.06
**Block 3**	R2 = 0.06 R2 adj = -0.05 ($F_{7,61}$ = 0.54, p = 0.799)						
	(Constant)		6.84	<0.001			
	Age	-0.11	-0.71	0.482	-0.09	[-0.33, 0.15]	1.44
	Education - years	-0.01	-0.05	0.964	-0.01	[-0.25, 0.24]	1.42
	BMI (kg/m2)	-0.10	-0.73	0.468	-0.10	[-0.33, 0.15]	1.28
	Days of CPAP	0.20	1.48	0.144	0.19	[-0.06, 0.41]	1.09
	ESS	-0.07	-0.48	0.630	-0.06	[-0.3, 0.18]	1.23
	%T90	0.08	0.58	0.567	0.08	[-0.17, 0.31]	1.08
	NfL (pg/mL)	0.03	0.20	0.839	0.03	[-0.22, 0.27]	1.30

Note. BMI: body mass index; %T90: percentage of total sleep time spent with oxygen saturation below 90%; Ess - Total: Epworth Sleepiness Scale total score; NfL: light chain neurofilament.
